# Metabolic Reprogramming in Kidney Diseases: Evidence and Therapeutic Opportunities

**DOI:** 10.1155/2021/5497346

**Published:** 2021-10-25

**Authors:** Yin Li, Zixin Sha, Hui Peng

**Affiliations:** ^1^Department of Nephrology, The Third Affiliated Hospital, Sun Yat-Sen University, Guangzhou, China; ^2^Department of Biological Sciences, Carnegie Mellon University, Pittsburgh, PA, USA

## Abstract

Metabolic reprogramming originally referred to the ability of cancer cells to metabolically adapt to changes in environmental conditions to meet both energy consumption and proliferation requirements. According to recent studies, renal cells are also capable of reprogramming their metabolism after kidney injury, and these cells undergo different kinds of metabolic reprogramming in different kidney diseases. Metabolic reprogramming also plays a role in the progression and prognosis of kidney diseases. Therefore, metabolic reprogramming is not only a prominent feature but also an important contributor to the pathophysiology of kidney diseases. Here, we briefly review kidney diseases and metabolic reprogramming and discuss new ways to treat kidney diseases.

## 1. Introduction

The idea of metabolic reprogramming first came from the Warburg effect in cancer cells. Despite the presence of sufficient oxygen, oxidative phosphorylation (OXPHOS) in mitochondria is inhibited, and cells tend to utilize glycolysis to produce energy. This altered metabolism was first recognized by the Nobel Prize winner Otto Warburg and is therefore termed the Warburg effect or aerobic glycolysis [1]. Although less efficient than OXPHOS, aerobic glycolysis provides sufficient energy for survival and the production of structural components [2]. For years, the mechanism and significance of the Warburg effect have been the centre of the cancer metabolism. However, metabolic reprogramming includes not only the Warburg effect but also other metabolic transformations to adapt to shifting environments. Metabolic reprogramming is also not only related to cancer. Emerging evidence suggests that it is also involved in kidney diseases. In this review, we present recent studies in this field that may offer new opportunities for the treatment of kidney diseases.

## 2. Autosomal Dominant Polycystic Kidney Disease

Autosomal dominant polycystic kidney disease (ADPKD) is the leading genetic disease associated with end-stage renal disease and is caused by loss-of-function mutations in either PKD1 or PKD2. The main characteristic of ADPKD is that excessive proliferation of epithelial cells results in the relentless expansion of cysts. Rowe et al. discovered that mouse embryonic fibroblasts (MEFs) isolated from Pkd1^−/−^ embryos acidified the medium faster than Pkd1^+/+^ cells, which suggested that mutations in Pkd1 result in a defective glucose metabolism. Moreover, enzymes involved in gluconeogenesis were decreased, while enzymes involved in glycolysis were increased, which was also found in murine models of PKD as well as human ADPKD kidneys. This study showed that PKD1^−/−^ cells in ADPKD mainly use aerobic glycolysis to supply energy and promote proliferation, which was the first time metabolic reprogramming had been observed in kidney disease. The researchers also found that 2-deoxyglucose (2DG), a glucose analogue that cannot be metabolized, depressed glycolysis and inhibited the proliferation of Pkd1^−/−^ cells, thereby reducing the cystic index [3].

Defective glucose metabolism is a prominent feature of ADPKD, and other metabolic pathways also change during ADPKD. Using the nontargeted global metabolism, Podrini et al. found that the loss of Pkd1 in the mouse kidney resulted in broad and coordinated metabolic reprogramming: glycolysis, the pentose phosphate pathway (PPP), fatty acid synthesis (FAS), and glutamine uptake increased, while the tricarboxylic acid (TCA) cycle and fatty acid oxidation (FAO) decreased [4]. Treatment of Pdk1 mutant mice with a glutamine inhibitor before birth slowed ADPKD progression [5, 6]. Pkd1^−/−^ cells preferably use glutamine to sustain the TCA and fatty acid biosynthesis. These cells utilize glutamine through asparagine synthase (ASNS), and so, inhibiting ANSN can reduce proliferation and increase apoptosis. The researchers found that targeting asparagine synthetase (ASNS) to interfere with glutaminolysis in conjunction with glycolysis could slow PKD1^−/−^ cell growth and survival. These findings suggest that the aerobic glycolysis pathway and PPP can increase the growth of cystic epithelial cells that exacerbate disease progression.

In addition, a decrease FAO can also exacerbate ADPKD. The transcription factor MYC reprograms the cell metabolism to maintain the rapid proliferation of ADPKD cells, similar to cancer cells. In ADPKD mouse models, c-MYC upregulates miR-17 in cystic kidneys, and miR-17 inhibits FAO by directly inhibiting PPAR*α* to reprogram the mitochondrial metabolism. The transcription factor PPAR*α* is involved in the regulation of the lipid metabolism. Suppressing miR-17 could restore PPAR*α* and improve FAO, ameliorating ADPKD [7, 8]. In addition, PPAR*α* agonist fenofibrate could increase PPAR*α* expression and FAO, reducing the cystic volume by 60% [9]. Therefore, targeting a combination of metabolic regulators may be a promising therapeutic approach in ADPKD.

## 3. Acute Kidney Injury

Acute kidney injury (AKI) can be caused by various factors, including ischaemic or hypoxic injury, infection, and toxins [10]. The corresponding animal models are the AKI model, the renal ischaemia-reperfusion injury model, the LPS-induced AKI model, and the nephrotoxicity model (cisplatin and radiocontrast agents). The key pathogenic factor of AKI is tubular epithelial cell (TEC) injury. TECs have high energy consumption levels and high baseline metabolic rates to continuously reabsorb urobilinogen components, such as water, amino acids, and glucose. The fatty acid metabolism is the main metabolic pathway in TECs, as this pathway produces energy most effectively. However, TECs are reprogrammed to use aerobic glycolysis in AKI [11]. One of the main causes of this phenomenon is mitochondrial damage; because mitochondria are the site of the fatty acid metabolism, proximal tubular cells shift to the glycolysis pathway to compensate for insufficient energy. Early in LPS-induced AKI, this shift in the metabolism is necessary for the development of trained immunity [12], and it is an effective response to injury in the early phase. However, a persistent proinflammatory state will worsen kidney function and prognosis. It is important to switch glycolysis back to OXPHOS to turn off inflammation. Several studies also suggest that depressing aerobic glycolysis and increasing OXPHOS could protect organs and improve survival rates [13, 14]. Zhou et al. further explored the specific molecular mechanism. The researchers showed that endothelial nitric oxide synthase (eNOS) could turn pyruvate kinase M2 (PKM2) into S-nitrosylated PKM2 (SNO-PKM2) through SNO-CoA. This transformation makes PKM2 unable to catalyse phosphoenolpyruvate, reducing glycolysis and increasing both the pentose phosphate pathway (PPP) and serine synthesis. Ultimately, such metabolic reprogramming increases the synthesis of lipids, proteins, and nucleotides, which promotes TEC repair and alleviates AKI. This finding indicates that eNOS can transform glucose utilization from energy production to tissue regeneration after AKI [15].

Another study showed that human umbilical cord mesenchymal stromal cells (UC-MSCs) could facilitate renal tubule repair in cisplatin-induced AKI. Cisplatin decreases the expression of TEC genes involved in mitochondrial energy production, including the amino acid metabolism, urea cycle, fatty acid metabolism, and electron transport chain components. UC-MSCs could repair and replenish mitochondria and increase the gene expression of electron transport chain components and proteins involved in generating ATP, enabling damaged TECs to reprogram their metabolism to sustain energy supplies [16]. Thus, UC-MSCs can protect TECs and drive regeneration following AKI.

If cells are unable to switch from aerobic glycolysis to OXPHOS [17] and FAO [18], renal fibrosis and further CKD may result [19].

## 4. Chronic Kidney Disease

Tubulointerstitial fibrosis is common in all end-stage chronic kidney diseases (CKDs) caused by various pathological changes. Major activators of fibrosis include the activation of renal intrinsic fibroblasts and transdifferentiation of TECs [20, 21]. Increased glycolysis in renal intrinsic fibroblasts and defects in the lipid metabolism in TECs are the main types of metabolic reprogramming in CKD progression.

An important feature of tubulointerstitial fibrosis is sustained activation of intrinsic renal fibroblasts. The preeminent fibrogenic cytokine TGF*β*1 induces a shift in renal myofibroblasts from OXPHOS to aerobic glycolysis and enhances the glutamine metabolism. Consequently, the metabolic switch to aerobic glycolysis reduces the expression of acetyl-CoA, which upregulates histone 3-related gene expression [22] and increases the expression of fibrotic genes [23]. In addition, the enhanced glutamine metabolism is needed to support the biosynthetic requirements of renal myofibroblasts [24]. This metabolic reprogramming is highly correlated with the development of renal interstitial fibrosis [25].

In studies of TECs, Kang et al. found that TGF-*β*1 impairs the renal tubular fatty acid metabolism through SMAD3 and PGC-1*α* and participates in tubulointerstitial fibrosis in a folic acid-induced nephropathy (FAN) mouse model. Restoring FAO by genetic or pharmacological intervention protected mice against tubulointerstitial fibrosis [18]. The team also found that the direct binding of Jag1/Notch2 and mitochondrial transcription factor A (Tfam) played a key role in reducing FAO and TEC transdifferentiation. Reexpression of Tfam in TECs prevented Notch-induced metabolic reprogramming and kidney fibrosis development [26]. Our study also showed a significant decrease in TEC oxygen consumption and dysfunctional lipid and glucose metabolisms in the FAN mouse model. We also found that exercise could counteract metabolic reprogramming and fibrogenesis through the myokine irisin [27].

Accordingly, the feasibility and efficiency of targeting renal lipid metabolism pathways to ameliorate fibrosis, including CD36, CPT1/2, PPARs, peroxisome proliferator-activated receptor-*γ* coactivator (PGC-1*α*), proprotein convertase subtilisin/kexin type 9 (PCSK9), and noncoding RNAs, have been explored in many preclinical experiments. Although clinical trials on these emerging regulators are still lacking, they represent promising strategies to prevent CKD progression [28].

## 5. Diabetic Kidney Disease

Diabetic kidney disease (DKD) is one of the main microvascular complications of diabetes, and it has become the leading cause of end-stage renal disease. Diabetes affects every kind of cell in the kidney, including podocytes, TECs, glomerular endothelial cells, and mesangial cells. Among them, podocytes and TECs play key roles in the pathogenesis of DKD [29, 30]. Diabetes elevates blood glucose and lipids, which leads to metabolic disorders and dysfunction [31, 32]. In the renal cortex in DKD, glycolysis and fatty acid metabolism increase to compensate for the loss of ATP in the TCA cycle. Moreover, the metabolism of glutamate and aspartate increases and the PPP decreases [33]. Some studies have shown that metabolic changes regulated by the lncRNA-mRNA coexpression network are associated with metabolic reprogramming in DKD [34, 35].

With respect to podocytes, a study published in Nature Medicine used proteomics analysis on glomeruli from patients with an extreme duration of diabetes (≥50 years) with DKD and without DKD and showed that seven out of the twelve top-ranked pathways were associated with the glucose metabolism and glycolysis. The researchers found that enzymes related to the podocyte glucose metabolism promoted the metabolism of excess intracellular free glucose and reduced the accumulation of toxic glucose products in cells, thereby protecting podocytes from hyperglycaemic toxicity. Furthermore, high glucose and diabetes reduced PKM2 tetramer formation, which impaired glycolysis. Podocyte-specific PKM2-knockout mice with diabetes exhibited worse albuminuria and glomerular pathology than wild-type mice, while pharmacological activation of PKM2 reversed the elevation in toxic glucose metabolites and mitochondrial dysfunction induced by high glucose, which protected against DKD [36].

Metabolic reprogramming in TECs also plays an important role in DKD pathogenesis. Kidney fibrosis in diabetes is associated with aberrant glycolysis in TECs. Excessive glycolysis in DKD is induced by SIRT3 deficiency through the induction of the TGF*β*-smad3 signalling pathway. SIRT3 deficiency transforms the PKM2 tetramer into a PKM2 dimer, which can translocate to the nucleus, promote the transcription of proglycolytic enzymes, and increase HIF1*α* and IL1*β* production. Inhibiting abnormal glycolysis disrupts metabolic reprogramming and suppresses fibrosis in DKD [37]. Although this conclusion seems contradictory, the former study focused on the active tetrameric form of PKM2, while the latter study discussed the glycolytic inactive dimeric form of PKM2, which can translocate to the nucleus to regulate gene expression and induce abnormal glycolysis. In addition, TECs are highly proliferative and primarily utilize fatty acids, while podocytes are nearly quiescent [38] and utilize anaerobic glycolysis [39]. This may account for differences in metabolic reprogramming in different cells under the same disease, depending on the basic metabolic types of these cells. This finding indicates that metabolic reprogramming in kidney disease is complex and diverse. Therefore, highly specific targeting in specific renal cells will be difficult, but will be key to future treatments.

## 6. Other Kidney Diseases

Different types of glomerulonephritis may share the same metabolic reprogramming. A study showed that in nephrotic syndrome (NS) and ANCA-associated vasculitis (AAV), gene expression related to the TCA cycle, FAO, and glutaminolysis in the glomerular compartment were repressed compared with those in the normal control, while the gene expression of PPP in NS and AAV was significantly increased relative to that in the normal control. Elevated expression of PPP factors was also observed in the tubulointerstitial compartment, and a significant negative association between PPP factor expression and GFP in both the glomerular compartment and the tubulointerstitial compartment was observed. PPP factor expression in the tubulointerstitial compartment was also shown to be associated with an increased degree of fibrosis. The study also suggested that renal monocytes/macrophages were likely major contributors to PPP factor expression in these kidney diseases [40].

The PPP can not only generate NADPH and maintain redox balance, which may be particularly important for cells undergoing oxidative stress, but also synthesize various cellular components. Activation of the PPP could promote T cell proliferation and induce cytokine production in both T cells [41] and macrophages [42]. A strong correlation was observed between the PPP and lymphocyte activation in SLE [43] and TNF activation in AAV [40]. Therefore, the same reprogramming of metabolic pathways in inflammatory cells might be shared across different types of glomerulonephritis, especially in patients with inflammatory kidney diseases [44].

## 7. Conclusion

**Figure 1:**
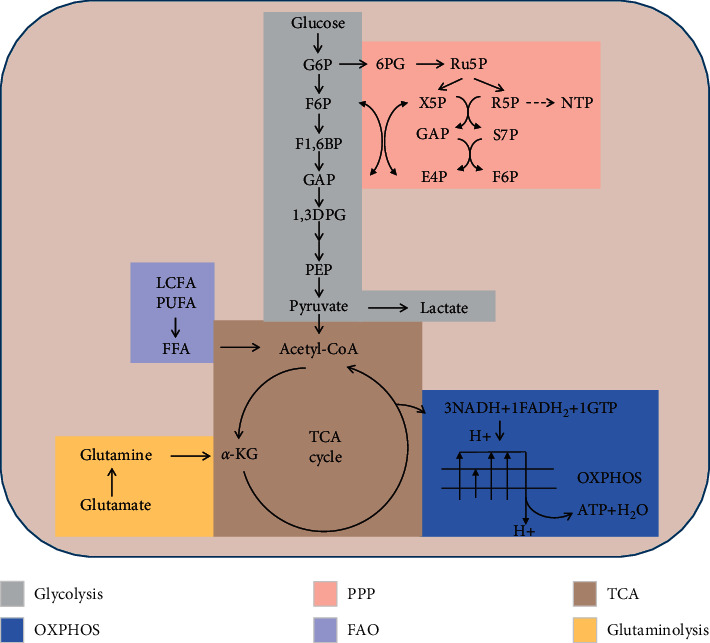
Significant metabolites colour-coded according to the pathway classification. Scheme of the glycolysis (GLY), pentose phosphate pathway (PPP), tricarboxylic acid (TCA) cycle, fatty acids oxidation (FAO), and glutaminolysis in the cell. All abbreviations are in Table 2.

According to these studies, metabolic reprogramming plays an important role in kidney diseases (Table 1). However, many of the cited studies have only examined mRNA levels or metabolite concentrations, which are not necessarily the same as a change in carbon flux (Figure 1 and Table 2). Therefore, future work should focus on this limitation to verify the role of metabolic reprogramming.

Metabolic reprogramming is not only a result of kidney disease progression but also affects the outcome and prognosis of kidney diseases. The kidney is composed of various types of cells, all of which exhibit different kinds of the baseline metabolism and metabolic reprogramming in different kidney diseases. Thus, rather than a simple change in the energy or glucose metabolism, metabolic reprogramming is an adaption mechanism specific to the type of renal cell and the disease. However, the full characteristics of the metabolism in all renal cell types have yet to be defined. Future research should focus on investigating the adaptions of specific metabolic pathways in kidney diseases. Moreover, the downstream effects of key molecules in metabolic reprogramming are unclear, and their functions and mechanisms need to be explored further to provide new targets for the early diagnosis and treatment of renal diseases. Understanding these mechanisms will help discovering the discovery of new therapeutic targets and create new opportunities for the treatment of kidney diseases.

## Figures and Tables

**Table 1:** Metabolic adaptations in various cell types of different kidney diseases.

Disease	Cell type	Metabolic adaptations	References
ADPKD	Epithelial cells	Glycolysis ↑	[3]
		PPP ↑	[4]
		Glutamine uptake ↑	[5, 6]
		FAO ↓	[7–9]

AKI	TEC	Glycolysis ↑	[11]
		OXPHOS ↓	[13, 14, 17]
		FAO ↓	[18]

CKD	Fibroblasts	Glycolysis ↑	[22]
		Glutamine metabolism ↑	[24]
	TEC	FAO ↓	[26, 27]
		OXPHOS ↓	[28]

DKD	Podocytes	Glycolysis ↓	[37]
	TEC	Glycolysis ↑	[38]

NS, AAV	Monocytes/macrophages	PPP ↑	[41]

SLE	Lymphocytes	PPP ↑	[44]

FAO, fatty acid oxidation; TEC, tubular epithelial cells; OXPHOS, oxidative phosphorylation; PPP, pentose phosphate pathway; NS, nephrotic syndrome; AAV, ANCA-associated vasculitis.

**Table 2:** Abbreviations.

*α*-KG	Alpha-ketoglutarate
1,3DPG	1,3-Diphosphoglycerate
6PG	6-Phosphogluconate
E4P	Erythrose-4-phosphate
FAO	Fatty acid oxidation
FFA	Free fatty acid
F6P	Fructose-6-phosphate
F1,6BP	Fructose-1,6-bisphosphate
G6P	Glucose-6-phosphate
GAP	Glyceraldehyde-3-phosphate
LCFA	Long-chain fatty acids
NTP	Nucleoside triphosphate
OXPHOS	Oxidative phosphorylation
PEP	Phosphoenolpyruvate
PPP	Pentose phosphate pathway
PUFA	Polyunsaturated fatty acid
R5P	Ribose-5-phosphate
Ru5P	Ribulose-5-phosphate
S7P	Sedoheptulose-7-phosphate
X5P	Xylulose-5-phosphate
